# Design and baseline characteristics of a prospective cohort study for determinants of osteoporotic fracture in community-dwelling elderly Japanese men: the Fujiwara-kyo Osteoporosis Risk in Men (FORMEN) Study

**DOI:** 10.1186/1471-2474-10-165

**Published:** 2009-12-24

**Authors:** Masayuki Iki, Yuki Fujita, Junko Tamaki, Katsuyasu Kouda, Akiko Yura, Eiko Kadowaki, Yuho Sato, Jong-Seong Moon, Nozomi Okamoto, Norio Kurumatani, Study Group for Functioning Capacity and Quality of Life in Elderly Japanese (Fujiwara-kyo Study Group)

**Affiliations:** 1 Department of Public Health, Kinki University School of Medicine, 377-2 Oono-higashi, Osaka-Sayama, Osaka 589-8511, Japan; 2 Department of Human Life, Jin-ai University, 3-1-1 Ootemachi, Echizen, Fukui 915-8586, Japan; 3 Faculty of Human Sciences, Taisei Gakuin University, 1030-1 Hirao, Mihara-ku, Sakai, Osaka 587-8555, Japan; 4 Department of Community Health and Epidemiology, Nara Medical University School of Medicine, 840 Shijocho, Kashihara, Nara 634-8521, Japan

## Abstract

**Background:**

Osteoporosis and osteoporotic fracture in men are significant public health problems in an aging society. However, information on male osteoporosis remains impressively lacking, especially for Asians. We designed the Fujiwara-kyo Osteoporosis Risk in Men (FORMEN) study as an ancillary study of a cohort study, the Fujiwara-kyo study, to determine risk factors for osteoporotic fractures in Japanese men.

**Methods/Design:**

***Design***: A community-based single-centre prospective cohort study with at least a 5-year follow-up

***Subjects***: All the male participants of the Fujiwara-kyo study who were living in the four cities studied, aged 65 years and older, and able to walk without aid from others.

***Primary outcome***: Incidence of osteoporotic fractures including vertebral and clinical non-vertebral fractures.

***Additional outcomes***: Change in bone mineral density (BMD), change in hip geometry, onset of receiving benefits from Long-term Care Insurance (LCI), health-related quality of life, and mortality.

***Baseline measurements***: BMD at the lumbar spine (LS) and hip (TH), hip geometry, vertebral deformity assessment, bone turnover markers, physical and cognitive performance, various medical and lifestyle factors, and geriatric psychosocial measures confirmed by interviews based on self-administrated questionnaires.

***Outcome surveillance***: Annual mail surveys and a follow-up survey at the fifth year comprising similar items to the baseline study will be used to determine the outcomes. Receipt of benefits from LCI and mortality will be obtained from the city governments.

***Current status***: The baseline study was conducted for 2174 eligible men, and 2012 completed the study and were eligible for follow-up. Prevalence rates of osteoporosis (BMD 2.5 SD or more below the young adult mean (YAM)) and low BMD (BMD 1 SD or more below YAM) in at least one of LS and TH were calculated to be 4.4% and 41.8%, respectively. The proportion of men with low BMD only at TH showed a significant increasing trend with aging (p < 0.0001) while that only at LS showed a decreasing trend (p = 0.0386). The prevalence rate of osteoporosis was underestimated when diagnosed using only BMD at LS. Other baseline measurements were successfully obtained.

**Discussion:**

FORMEN baseline study was performed as designed and the FORMEN cohort study was successfully launched.

## Background

Osteoporotic fracture is a significant and increasing public health burden, for men as well as women, in aging societies. The lifetime risk for osteoporotic fracture was estimated to be 40% in women and 13% in men [1]. These figures, however, may be underestimated by nearly half, and have been suggested to be as high as 23% in men when taking future reduction of mortality into account [2]. The incidence of hip fracture, the most serious osteoporotic fracture, is increasing. The number of hip fractures is projected to be 13 million in 2050, with 31% of these (approximately 4 million), occurring in men [3]. It has been well established that the incidence rate of hip fracture is much higher in Caucasians than in Asians [4], but Asians will account for 45% of hip fractures globally in 2050 because the population of elderly people in Asia is increasing dramatically [3]. In Japan, nation-wide surveys for the incidence of hip fracture have been conducted four times since 1987 [5]. There were 117,900 incident hip fractures estimated in 2002, double the number from 1987, of which 22% occurred in men [6]. This trend in the incidence of osteoporotic fractures urges us to establish a comprehensive strategy to prevent and manage osteoporosis in men as a national and international issue with the highest priority.

In spite of the magnitude of the problem posed by osteoporosis in men, there remains a substantial shortage of information concerning male osteoporosis, especially in Asia. Only a few prospective cohort studies have been performed for the determinants of osteoporotic fracture in Hong Kong Chinese men [7] and in Japanese men [8-10]. Some of these have been well performed with a sufficiently high follow-up rate, but the sample size did not exceed 800, which was not sufficiently large for evaluating determinants of osteoporotic fractures [8-10]. Furthermore, comprehensive geriatric assessments for the evaluation of risk factors for osteoporotic fracture are lacking. Without these data, it would be difficult to design rational clinical and preventive approaches for osteoporosis and osteoporotic fracture in men. Thus, we have designed and launched the Fujiwara-kyo Osteoporosis Risk in Men (FORMEN) Study, a large-scale community-based single-centre prospective cohort study for elderly Japanese men, to address the following questions:

1. Does bone mass predict fracture risk in men as has previously been demonstrated in women?

2. Which lifestyle or medical factors are associated with fracture risk in men?

3. Do physical performance test results predict fracture risk in men?

4. Do bone turnover markers enhance the predictive value of bone density and other clinical risk factors for fracture risk in men?

5. Does socioeconomic status affect the risk of osteoporosis and osteoporotic fracture in men?

6. To what extent do fractures in men increase the risk of being dependent on care provided from Long-term Care Insurance (LCI)?

7. To what extent do fractures affect quality of life (QOL) in men?

8. Do existing vertebral fractures and incident non-vertebral fractures increase risk of death in men?

We present in this paper the design of the FORMEN study and the baseline characteristics of its participants.

## Methods/Design

### General study description

The FORMEN study is an ancillary study of a larger prospective cohort study, The Cohort Study for Functioning Capacity and Quality of Life in Elderly Japanese (Primary Investigator: Norio Kurumatani, M.D., Ph.D., Professor and Chairman, Department of Community Health and Epidemiology, Nara Medical University School of Medicine), which is referred to as the "Fujiwara-kyo study" after the study area where the first capital of Japan, called "Fujiwara-kyo," was established in 694 AD. The Fujiwara-kyo study aims to provide a scientific basis for a comprehensive strategy to prevent frailty, increase the number of healthy life years, and promote the functioning capacity and quality of life of the elderly men and women in Japan. The FORMEN study examines male participants of the Fujiwara-kyo study for bone health. Most of the non-skeletal measurements and interviews were conducted under the Fujiwara-kyo study, and these data form a comprehensive geriatric and gerontologic basis for the FORMEN study to evaluate risk factors of osteoporosis and osteoporotic fracture of elderly men. Brief explanations of the Fujiwara-kyo study appear in this paper and details of the study are described elsewhere. (Kurumatani N, Okamoto N, Morikawa M, Iwamoto J, Hazaki K, Harano A, Tanaka K, Minematsu A, Tomioka K, Saeki K, Amano N, Yanagi M: Protocol and baseline data of a prospective cohort study (Fujiwara-kyo study) on the change of functioning capacity and quality of life among physically independent elderly people: A report from a world's top longevity country, Japan, submitted)

### Study population and recruitment of samples

**Figure 1:**
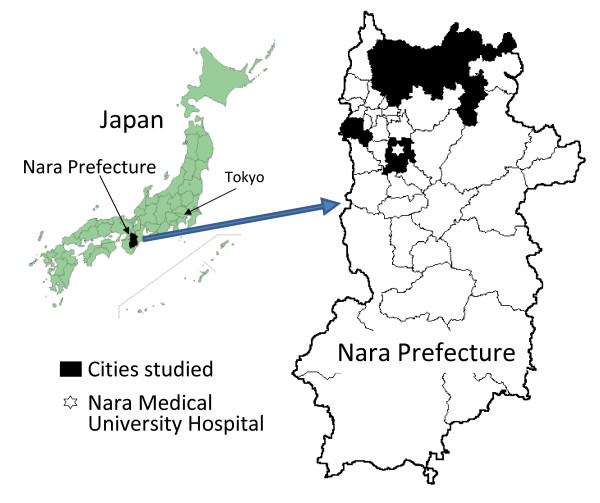
**Study area of the Fujiwara-kyo study**.

To achieve the aims of the Fujiwara-kyo study, participants in the study should be representative of elderly men and women who are still living independently in a community and can participate in various preventive activities conducted by their municipalities. For the inclusion criteria, therefore, participants should be:

(1) aged 65 years or older at baseline,

(2) living in their homes in the cities of Kashihara, Nara, Yamato-Kooriyama, and Kashiba, which are surrounding areas of the Nara Medical University Hospital (Figure 1),

(3) able to walk without the assistance of another person,

(4) able to provide self-reported information, and

(5) able to understand and provide written informed consent.

The FORMEN study included all the male participants of the Fujiwara-kyo study.

The recruitment of participants was conducted by the Administrative Centre of the Fujiwara-kyo study with the cooperation of local residents' associations and elderly people's clubs organized in every district of the cities studied. These groups used printed literature that included information on the aims of the study, study procedures, risks and benefits posed by study measurements, rights and responsibilities of participants and researchers, publication of results, and protection of privacy. The participants were asked to give written informed consent before enrolment in the study.

The sample sizes necessary for the Fujiwara-kyo study and the FORMEN study were set as 4500 and 2000 as described later, respectively.

### Outcome indices

The primary outcome for the FORMEN study is the incidence of osteoporotic fractures, including vertebral fractures and clinical non-vertebral fractures. The additional outcomes consist of change in bone mineral density (BMD), change in hip geometry, onset of receiving benefits from LCI, which is an objectively assessed index of having become physically or cognitively dependent on other people in daily life, change in QOL, and death.

### Study measures

#### Skeletal measures

##### Bone mass measurement

Bone mineral content (BMC) (g) and bone area (cm^2 ^) were measured by dual-energy X-ray absorptiometry (DXA) at the lumbar spine (L2-4) and right hip in a posteroanterior projection (QDR4500A, Hologic Inc., Bedford, MA, USA) as previously described [11]. Participants with either a history or incident involvement of fractures or bone disease in the right hip were scanned on the left side. We excluded densitometric data of the spine for participants with vertebral fractures or grade four osteophytes according to Nathan's classification [12], or those with hip deformities in the regions of interest (ROI), from the analysis. All spine and hip measurements throughout the study were made with one scanner installed in a mobile test room in a large vehicle. BMD (g/cm^2 ^) was obtained by BMC divided by bone area at the lumbar spine (LS) and total hip (TH). TH was divided into several regions including the femoral neck (FN) and trochanter (TR). The short-term precision as measured by the coefficients of variation (CV) of the BMD measurements in vivo was 1.2%, 1.2%, 1.6% and 1.9% for LS, TH, FN and TR, respectively. Quality assurance of the measurements was conducted daily before the study measurements began. We observed no remarkable drift in BMD values for the spine phantom throughout the baseline study period.

We defined osteoporosis as BMD values 2.5 SD or more below the young adult mean (YAM) [13], and low BMD as 1 SD or more below YAM, in accordance with the WHO criteria [14].

##### Hip geometric assessment

The archived DXA image used for calculating BMD at the hip was subsequently analyzed using Apex software's Hip Structure Analysis (HSA) program (Hologic Inc., Bedford, MA, USA). The precise method has been described elsewhere [15]. The HSA program automatically set three ROIs defined as: (1) narrow neck, traversing the narrowest width of the femoral neck, (2) intertrochanter, along the bisector of the shaft and femoral neck axes, and (3) shaft, at 1.5 times the minimum neck width distal from the intersection of the neck and shaft axes. The HSA program yielded several hip geometric indices including cross-sectional area, cross-sectional moment of inertia, section modulus, cortical thickness and subperiosteal diameter for each of three ROIs.

##### Vertebral deformity assessment

We imaged the thoracolumbar vertebrae of each participant by single-energy X-ray absorptiometry (SXA) following bone density measurements. Bone morphometric software (QDR4500A Lateral Image Analyze, Hologic Inc., Bedford, MA, USA) was used to measure anterior, central and posterior edge heights of each vertebra using the SXA images. We diagnosed vertebral deformities according to the McCloskey-Kanis criteria [16] and used these as a proxy for vertebral fracture in the baseline study. We visually confirmed all deformed vertebrae according to Genant's semiquantitative assessment method [17].

##### History of symptomatic fractures

History of symptomatic fracture was determined by interviewer-confirmed self-reports from participants. When a participant reported a symptomatic fracture having occurred at any time of his life, a trained registered nurse conducted an in-person interview to determine when the injury occurred, which bone or part of body was injured, what activities the participant was engaged in when injured, and whether a physician diagnosed the injury as a fracture according to radiographic examinations. We defined a symptomatic fracture as one which caused a clinic visit due to pain and was diagnosed as a fracture by a physician using radiographs.

##### Bone turnover markers and other laboratory measures

We drew blood from each participant after an overnight fast and obtained serum for several conventional biochemical tests planned in the Fujiwara-kyo study. We stored the remaining serum at -80°C until we performed measurements for the FORMEN study. We measured levels of bone turnover markers including osteocalcin (OC), undercarboxylated OC (ucOC), and tartrate-resistant acid phosphatase isoenzyme 5b (TRACP-5b). In addition, we will measure Type I collagen cross-linked C-terminal telopeptide (CTX) and type I procollagen N-terminal propeptide (P1NP). As non-skeletal markers, we measured serum levels of high-sensitivity CRP and homocysteine.

We measured OC [ng/ml] using a two-site immunoradiometric assay (BGP IRMA kit Mitsubishi, Mitsubishi Kagaku Iatron Inc., Tokyo, Japan) with a sensitivity of 1 ng/ml [18]. The precision of this measurement was 4.9%, 3.7% and 6.1% for intraassay CV, interassay CV and overall CV, respectively.

We used an electrochemiluminescence immunoassay to measure ucOC [ng/ml] (Picolumi ucOC, Sanko Junyaku Co. Ltd., Tokyo, Japan) with a sensitivity of 0.39 ng/ml [19]. The precision of this measurement was 4.1%, 3.5% and 5.4% for intraassay CV, interassay CV and overall CV, respectively.

TRACP-5b was measured by a fragment-absorbed immunocapture enzyme assay (Osteolinks-TRAP-5b, Nitto Boseki, Kooriyama, Japan) with a sensitivity of 19.2 mU/dl [20]. The intraassay CV, interassay CV and overall CV of this measurement in our laboratory were 4.9%, 7.3% and 8.8%, respectively.

Serum levels of CTX and P1NP will be measured using an enzyme-linked immunoassay (Serum CrossLaps ELISA, Immunodiagnostic Systems Ltd., Boldon, UK)[21] and a two-site electrochemiluminescence immunoassay (Roche Elecsys 2010, Roche Diagnostics, Lewes, UK)[22], respectively.

#### Non-skeletal measures

Non-skeletal measures were obtained in the Fujiwara-kyo study. We state briefly here some measures relevant to the FORMEN study.

##### Clinical measures

We measured participant height (cm), weight (kg) and waist circumference (cm), and calculated body mass index (BMI)(kg/m^2 ^).

##### Physical and cognitive performance measures

A series of physical performance tests for elderly people were conducted including grip strength, muscle strength of knee extensors and flexors, brisk gait speed, the timed up-and-go test, and the timed one-leg balance with eyes open. To quantify cognitive function, participants completed the Modified Mini-Mental State Examination [23].

##### Medical history, and gerontological lifestyle and socioeconomic factors

Participants completed a questionnaire including 250 items covering medical history including thyroid diseases, parathyroid diseases, rheumatoid arthritis, connective tissue diseases, malignant diseases, hypertension, diabetes mellitus, coronary heart diseases, hyperlipidemia, asthma, kidney diseases and prostate diseases, history of medications due to these diseases, several scales of geriatric and gerontological factors, smoking and drinking history, diet history including intake of dairy products and a fermented soy bean product called 'natto' in Japanese, physical activity indices necessary for quantification using the International Physical Activity Questionnaire (IPAQ) [24], fall and fracture history, family history of fractures, symptoms in back and joints, level of education, social and economic status, and marital status. Participants were asked to bring current prescriptions of medications to the baseline visit where interviewers recorded the name and dose of the medications. Participants were also asked to complete a seven-day diary of their diets to estimate dietary nutrient intakes.

##### Quality of life assessment

Health-related quality of life was assessed with the 36-item short form of the Medical Outcomes Study Questionnaire (SF-36) [25].

### Outcome surveillance

To obtain outcome indices, annual mail surveys and a follow-up survey in the fifth year comprising similar study items to the baseline study will be conducted as part of the Fujiwara-kyo study.

#### Incident vertebral fractures

An incident vertebral fracture is diagnosed morphometrically based on SXA images of the spine when the anterior, central or posterior height of a vertebra is reduced by 20% or more on follow-up compared to baseline, and when the vertebra also satisfied the McCloskey-Kanis criteria [16]. Vertebrae with heights reduced by 20% or more which do not satisfy the McCloskey-Kanis criteria are considered to be incident fractures if they satisfy the definition of grade 2 or 3 fracture by the Genant method [17].

#### Change in bone density and hip geometry

Bone densitometry will be conducted in the follow-up survey at the same skeletal sites with the same densitometer as used in the baseline study. Changes in BMD at LS, TH, FN and TR, and in hip geometry indices will be determined.

#### Non-vertebral and symptomatic vertebral fractures

When a participant reports on the annual questionnaire the occurrence of fracture in the previous year, a trained nurse will conduct a telephone interview and obtain details of the injury to determine whether the injury is a fracture or not. Reviews of participant medical records will be further conducted with the written permission of the participant.

#### Onset of receiving benefits from LCI and death of participants

We will obtain information regarding whether a participant starts receiving benefits from LCI from response to the annual questionnaire. In the case that a participant dies in the previous year, the family is to inform the Administrative Centre of the Fujiwara-kyo study of the participant's death. When a participant does not respond to the annual questionnaire, the Administrative Centre will contact the city office and obtain the information on the participant's receipt of benefits and survival. Participants have given the researchers their consent to this inquiry.

### Statistical analysis

#### Primary analyses

The primary analyses consist of assessments of baseline risk factors for the primary and additional outcomes of the study. The risk factors will be thoroughly investigated from among the skeletal and non-skeletal measures obtained at baseline. To evaluate risk factors for vertebral fractures and clinical non-vertebral fractures, we will conduct logistic regression analysis and Cox proportional hazards regression analysis, respectively. Factors associated with changes in BMD and hip geometry indices will be evaluated by multiple regression analysis. The proportional hazards regression will also be applied to evaluate risk factors for receiving benefits from LCI, and mortality. Determinants for changes in QOL will be evaluated by multiple regression analysis. Additional effects of the incidence of osteoporotic fractures during follow-up on receiving LCI benefits, changes in QOL and survival will be evaluated after adjusting for their potential risk factors at baseline.

#### Additional analyses

Additional analyses, mostly cross-sectional, are being conducted for provisional assessments of known and potential risk factors for the outcome indices. These include the effects of socioeconomic status, medical histories, physical and cognitive performance, and lifestyle factors including smoking, drinking, milk consumption and "natto" intake on BMD, low BMD, and vertebral fractures.

### Sample size calculations

The sample size necessary for the FORMEN study was set as 2000. This was obtained in order that the logistic regression analysis yields a significant estimate with a two-tailed level of significance at 5% and a statistical power of 80% for a risk factor of fracture which exists in 20% of the population and increases the fracture risk by 75% compared with people without the risk factor who run the absolute fracture risk of 1% per year. The statistical power afforded by the sample size of 2000 under several other assumptions is shown in Table 1.

**Table 1:** Statistical power expected for logistic analysis under a sample size of 2000 with various assumptions.

Risk factor studied		Annual incidence rate of fracture (%) in population without a risk factor studied				
			
Prevalence rate (%)	RR	0.8	1	1.5	2	PARP (%)
20	1.25	14	18	24	41	4.8
	1.5	41	50	68	**81**	9.1
	1.75	72	**81**	**94**	**99**	13.0
						
	2	**91**	**96**	**100**	**100**	16.7

30	1.25	17	21	30	38	7.0
	1.5	43	59	78	**89**	13.0
	1.75	**80**	**88**	**98**	**100**	18.4
	2	**95**	**98**	**100**	**100**	23.1

40	1.25	19	23	32	42	9.1
	1.5	53	63	**81**	**92**	16.7
	1.75	**83**	**91**	**98**	**100**	23.1
	2	**96**	**99**	**100**	**100**	28.6

Statistical power was estimated for a logistic regression analysis to yield a significant estimate for a risk factor with a two-tailed level of significance at 5% under a sample size of 2000 with various combinations of assumptions.

RR: Relative risk of fracture posed by a risk factor studied.

PARP: Population attributable risk proportion.

**Boldface**: Power > 80%

81: Assumptions used when the present sample size of 2000 was determined.

### Study time line

**Figure 2:**
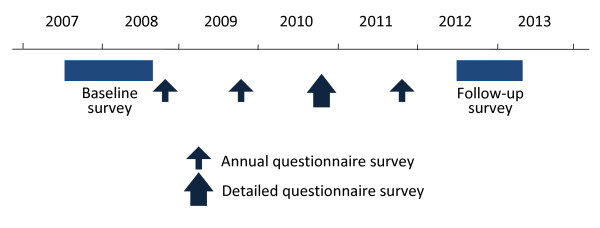
**Time line of the Fujiwara-kyo study**.

The complete schedule of the Fujiwara-kyo study is illustrated in Figure 2. The study enrolled participants and completed the baseline survey from June 2007 to October 2008. A follow-up survey covering most of the items examined in the baseline survey is scheduled in 2012 after an average follow-up period of 4.5 years. During the interim, the participants are asked to respond to annual mailed questionnaires for the surveillance of the outcome indices of the study. The third annual questionnaire covers more extensive items to update important information obtained at baseline.

### Study administration

The Administrative Centre of the Fujiwara-kyo study is organized in the Department of Community Health and Epidemiology, Nara Medical University School of Medicine. The Core Member Committee, established as a steering committee of the study, is chaired by the Principal Investigator and composed of core investigators from all the study areas. This committee takes responsibility for designing the primary scope of the study, developing protocols, organizing subcommittees for ancillary studies, setting specific research directions and goals, sharing publication opportunities, and smoothing inter-institutional collaborations. Core investigators are responsible for designing protocols for their specific area of study, conducting the measurements at the surveys, and collecting and collating the data for their areas of responsibility.

The FORMEN Administrative Center is organized in the Department of Public Health, Kinki University School of Medicine, to conduct the FORMEN study smoothly in close cooperation with the Fujiwara-kyo study.

The study protocol of the Fujiwara-kyo study was approved by the Medical Ethics Committee of the Nara Medical University, and that of the FORMEN study was approved by the Ethics Committee of the Kinki University School of Medicine.

### Current status

#### Participant recruitment

During the baseline study period, 2174 men and 2253 women participated in the Fujiwara-kyo study. Among the male participants, 2012 men completed the measurements conducted in the FORMEN study and were eligible for further analyses and the cohort study. The average age of the participants was 73.3 ± 5.3 (mean ± SD) years with a range of 65 to 94 years. They were 162.8 ± 5.7 cm tall, weighed 60.9 ± 8.6 kg, and had a mean BMI of 22.9 ± 2.8.

Table 2 shows comparisons of age distribution and body size parameters between FORMEN study participants and the male population of Nara Prefecture[26,27] or Japan [26,28]. The FORMEN participants comprised a higher proportion of men aged 70-79 and were slightly taller than the general population of Japan, but were very similar in body size to men in Nara Prefecture.

**Table 2:** Age and body size between FORMEN participants and male population of Nara Prefecture or Japan

Index	Population	65-69 years	70-74 years	75-79 years	80 years and older
Age	FORMEN study	627 (31.2%)	705 (35.0%)	450 (22.4%)	230 (11.4%)
distribution	Nara Prefecture^a^	34.7%	28.0%	20.3%	16.9%
	Japan^a^	33.2%	28.1%	20.6%	18.1%

Height (cm)	FORMEN study	164.8 ± 5.9	163.2 ± 5.9	162.7 ± 6	160.5 ± 6.4
	Nara Prefecture^b^	165.5 ± 6.2	164.1 ± 6.3	162.3 ± 6.3	160.5 ± 6.8
	Japan^c^	163.7 ± 6.5	162.2 ± 6.9	161.0 ± 6.3	158.9 ± 8.0

Weight (kg)	FORMEN study	63.3 ± 9.1	62.0 ± 8.6	60.0 ± 8.6	56.8 ± 8.8
	Nara Prefecture^b^	64.1 ± 9.2	62.8 ± 9.5	60.1 ± 9.2	56.6 ± 9.9
	Japan^c^	64.4 ± 9.7	62.0 ± 8.7	59.7 ± 8.4	56.0 ± 9.6

BMI (kg/m^2^)	FORMEN study	23.7 ± 3.2	23.7 ± 3.1	23.1 ± 3.3	22.4 ± 3.3
	Nara Prefecture^b^	23.8 ± 3.3	23.8 ± 3.4	23.2 ± 3.5	22.4 ± 3.6
	Japan^c^	24.4 ± 3.6	24.0 ± 3.4	23.4 ± 3.1	22.6 ± 3.5

^a ^Population Statistics based on the Basic Resident Registry [26]

^b ^Annual Report of Basic Health Examinations 2007 [27]

^c ^National Health and Nutrition Survey 2006 [28]

#### Characteristics of participants

##### Medical history and lifestyle factors

Medical history and lifestyle factors of the study participants are listed in Table 3. The prevalence rate of cerebrovascular diseases and malignant diseases including prostate cancer increased with aging. Lifestyle factors listed in this table were all significantly related to age. With the increase in age, participants drank more milk, smoked less, drank less alcohol and expended less energy. We also obtained information regarding other lifestyle factors, including dietary intake and exercise history, which will be presented in future publications.

**Table 3:** Baseline characteristics of participants stratified by age in the FORMEN baseline study, 2007-2008

	All age groups	65-69 years	70-74 years	75-79 years	80 years and older	P value ^a^
Past history and present involvement of diseases (%)						
Thyroid diseases	1.4	1.3	1.3	0.7	3.9	0.0915
Rheumatoid arthritis and connective tissue diseases	1.0	1.0	0.9	0.7	2.2	0.3419
Diabetes mellitus	10.1	9.4	10.7	10.9	8.7	0.9221
Asthma	1.2	1.6	0.7	1.3	1.3	0.7719
Hypertension	33.3	30.2	35.0	35.6	31.7	0.2787
Cerebrovascular diseases	3.7	2.6	2.8	6.4	3.9	0.0137
Coronary heart disease	4.7	4.3	4.6	4.9	5.7	0.4090
Malignant diseases	8.2	6.2	0.4	9.1	11.3	0.0115
Prostate cancer	2.4	1.6	2.1	3.8	3.0	0.0391
Steroid use	0.9	1.1	0.6	1.1	1.3	0.7588
Statin use	4.9	4.8	5.4	3.1	7.0	0.8217
Fragility fractures since age 50	4.4	1.2	4.4	4.9	4.4	0.7019
Lifestyle factors						
Milk consumption (%)						
2 glasses/day or more	8.1	6.2	8.4	9.1	10.0	0.0088
1 glass/day	43.2	40.4	43.9	40.4	53.9	
Several glasses/week	15.2	16.6	15.5	16.2	8.7	
1 glass/week or less	33.5	36.7	32.3	34.2	27.4	
Smoking status (%)						
Current smokers	17.0	21.9	17.3	14.3	7.9	<0.0001
Ex-smokers	61.4	56.6	59.8	65.1	71.9	
Non-smokers	21.7	21.5	23.0	20.8	20.2	
Number of pack-years in ever-smokers	41.1 ± 28.3	40.8 ± 28.5	43.2 ± 27.8	39.2 ± 27.8	39.9 ± 29.5	0.3908
Alcohol drinking habit (%)						
Less than once/week	38.0	33.3	37.8	40.9	45.8	0.0218
1-2 times/week	5.3	5.3	4.7	5.8	6.2	
3-5 times/week	8.9	10.4	8.9	7.4	7.5	
6 times/week or more	47.9	51.0	48.6	45.9	40.5	

Values represent mean ± SD, median with interquartile limits in parentheses, or proportion.

^a ^A trend of each variable with aging was tested by a linear regression-based trend test or Cochran-Armitage trend test. Chi-squared test was applied when a variable had more than two categories.

##### Skeletal measures

Skeletal measures of the study participants are shown in Table 4, stratified by age. BMD and BMC at LS were not significantly correlated with age, while those at TH, FN and TR were. Bone area did not clearly show an association with age. When adjusted for height and weight, BMD at TH still decreased with age (partial regression coefficient of -0.016 [SE 0.005] g/cm^2 ^/10 years [p < 0.0001]), but BMD at LS increased significantly (0.042 [0.008] g/cm^2 ^/10 years [p < 0.0001]). The levels of biochemical markers, such as OC, ucOC, and TRACP-5b increased with aging as well.

**Table 4:** Skeletal measures of participants stratified by age in the FORMEN baseline study, 2007-2008

Index	All age groups	65-69 years	70-74 years	75-79 years	80 years and older	P for trend
BMD (g/cm^2 ^)						
LS	1.026 ± 0.191	1.022 ± 0.177	1.023 ± 0.185	1.030 ± 0.199	1.042 ± 0.226	0.1877
TH	0.878 ± 0.126	0.901 ± 0.124	0.880 ± 0.121	0.864 ± 0.129	0.841 ± 0.130	<0.0001
FN	0.740 ± 0.116	0.763 ± 0.117	0.741 ± 0.111	0.720 ± 0.114	0.715 ± 0.118	<0.0001
TR	0.684 ± 0.112	0.702 ± 0.109	0.685 ± 0.107	0.674 ± 0.117	0.651 ± 0.119	<0.0001

BMC (g)						
LS	54.3 ± 13.3	54.2 ± 12.1	54.2 ± 13.4	54.5 ± 14.1	54.4 ± 14.4	0.7020
TH	36.5 ± 6.1	37.4 ± 6.3	36.5 ± 5.8	36.0 ± 5.9	35.2 ± 6.3	<0.0001
FN	3.9 ± 0.6	4.0 ± 0.6	3.9 ± 0.6	3.8 ± 0.6	3.8 ± 0.7	<0.0001
TR	8.9 ± 2.5	9.2 ± 3.5	8.9 ± 1.8	8.8 ± 1.8	8.5 ± 2.0	<0.0001

Area (cm^2 ^)						
LS	51.9 ± 5	52.2 ± 4.6	51.8 ± 5.1	52.0 ± 5.1	50.9 ± 5.2	0.0039
TH	41.6 ± 3.5	41.5 ± 3.6	41.5 ± 3.4	41.7 ± 3.3	41.8 ± 3.6	0.1959
FN	5.3 ± 0.4	5.2 ± 0.3	5.3 ± 0.4	5.3 ± 0.3	5.3 ± 0.3	0.0801
TR	13.0 ± 2.0	13.1 ± 2.7	13.0 ± 1.5	13.0 ± 1.5	13.1 ± 1.6	0.6968

Biochemical marker of bone turnover						
OC (ng/ml)	4.8 (3.2, 6.5)	4.5 (2.6, 6.1)	4.8 (3.2, 6.5)	5.0 (3.5, 6.8)	5.4 (4.0, 6.9)	<0.0001
ucOC (ng/ml)	2.9 (1.5, 5.4)	2.7 (1.5, 4.9)	2.9 (1.6, 5.4)	3.0 (1.6, 5.7)	3.4 (1.8, 6.4)	<0.0001
TRACP-5b (mU/dl)	212.3 (121.7, 370.4)	200.2 (115.0, 348.7)	211.0 (120.2, 370.5)	212.3 (122.0, 369.3)	254.0 (152.5, 423.3)	<0.0001

BMD: Bone mineral density

BMC: Bone mineral content

LS: Lumbar spine TH: Total hip FN: Femoral neck TR: Trochanter

OC: Osteocalcin

ucOC: Undercarboxylated osteocalcin

TRACP-5b: Tartrate resistant acid phosphatase isoenzyme 5b

Values of bone densitometric measures represent mean ± SD.

OC values represent median with interquartile limits in parentheses.

ucOC and TRACP-5b values represent geometric mean with values for M-SD and M+SD in parentheses.

Bone densitometric data at the spine and at the hip were obtained from 1916 and 2008 participants, respectively, without severe deformities in the region of interest.

Number of participants with available measurements for OC, ucOC and TRACP-5b were 1971, 1970 and 2003, respectively.

**Figure 3:**
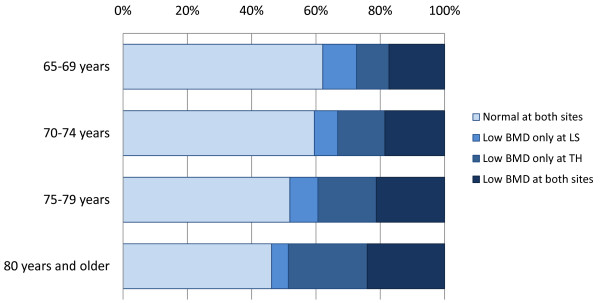
**Proportions of BMD categories in age-stratified participants of the FORMEN baseline study, 2007-2008**. BMD: Bone mineral density. Normal: BMD greater than 1 SD below the young adult mean (YAM). Low BMD: BMD 1 SD or more below YAM. LS: Lumbar spine. TH: Total hip. Data of 1912 subjects with BMD values available for both skeletal sites were used in the analysis.

The prevalence rates of osteoporosis in the FORMEN participants were 3.5% at LS, 1.9% at TH, and 4.4% in at least one of LS and TH. The prevalence rates of low BMD were 26.4%, 33.9% and 41.8%, respectively. Figure 3 illustrates proportions of low BMD categories in the participants in 10-year age groupings. The proportion of men whose BMD was judged to be low only at TH showed a significant increase with increase in age (p < 0.0001), while low BMD only at LS showed a decreasing trend with increasing age (p = 0.0386). Diagnosis of low BMD was not well correlated with BMD at LS and TH, with a kappa coefficient of 0.45. Other skeletal measures, including diagnosis of vertebral deformities and hip geometry indices, are being analyzed and will be presented in future papers.

##### Non-skeletal measures

Physical performance tests and cognitive performance tests were carried out successfully in most participants. The database is under construction and the results will be presented in future reports. We were also successful in obtaining QOL indices.

## Discussion

The FORMEN study is a large-scale community-based single-centre study for elderly Japanese men with comprehensive geriatric and gerontologic assessments for potential risk factors of osteoporosis and osteoporotic fractures. We have successfully conducted the baseline study as designed, and will carry out longitudinal observations for at least five years as a prospective cohort study.

The FORMEN study has some strengths compared with previous studies. First, the sample size of the FORMEN study is the largest in Japan, allowing for a high enough statistical power to evaluate risk factors of vertebral or osteoporotic fractures. Second, it has a comprehensive coverage of skeletal measures relevant to osteoporosis assessment, including vertebral deformity assessment and HSA in addition to conventional BMD measurement. Third, biochemical markers of bone turnover in sera were measured for all participants, and the remaining sera are stored at -80°C for further measurements of newer markers. Fourth, it has comprehensive coverage of potential risk factors of osteoporosis and osteoporotic fractures based on geriatric and gerontologic assessments. Fifth, we have established highly reliable surveillance of outcome indices using official municipality databases, including onset of becoming dependent on others in daily life and mortality. Finally, a single-centre study design has an advantage over a multi-centre design with respect to quality control of study performance given that no inter-centre calibrations of study measurements are necessary.

The FORMEN study also has potential limitations. First, participants were not randomly selected, which makes generalizing the results obtained difficult. However, the Fujiwara-kyo study aims to apply the results to people living independently in the communities studied, and our results can be utilized for this purpose. Second, the sample size of the FORMEN study may still be small for assessing risk factors of hip fracture. Third, BMD measurements did not include whole body BMD or volumetric BMD obtained by quantitative computed tomography. Fourth, vertebral deformity detected with SXA images might underestimate the prevalence of vertebral fractures compared to diagnosis with conventional radiographs. Fifth, urinary levels of bone turnover markers were not measured, although more novel serum markers were included. Finally, the deaths of participants will be confirmed, but a survey for the cause of death is not included in the initial study protocol.

Such potential limitations of the FORMEN study may have some effects on the study results, but are not expected to invalidate the results. In turn, they may suggest some directions for future research. The study items of the FORMEN study cover most items of preceding studies, such as the Mr OS study [29]. It may be possible to combine the results with those from other studies to increase the statistical power. Extending the follow-up period of the FORMEN study is worthwhile, and can increase the power as well. New random sample studies may be desirable to generalize study results to the whole population.

## Conclusions

The FORMEN study, a large-scale community-based single-centre prospective cohort study for elderly Japanese men with comprehensive coverage of potential risk factors of osteoporosis and osteoporotic fractures, designed as an ancillary study of the Fujiwara-kyo study, was successfully launched for longitudinal observations for at least five years. The study is expected to clarify a range of risk factors for osteoporotic fractures, to form predictive models of fracture risk, and to assess consequences of fractures in Japanese elderly men. These results should help health care professionals improve their preventive activities and management of osteoporosis in men.

## Competing interests

The authors declare that they have no competing interests.

## Authors' contributions

The Fujiwara-kyo Study Group performed most non-skeletal measures in the present study and provided the data to the FORMEN study. MI is the director of the FORMEN study, conceived of and designed the study, conducted the surveys and drafted the manuscript. YF is responsible for the FORMEN Administrative Center and conducted surveys in the study. JT participated in designing the study and conducting the surveys. YS analyzed DXA scan data to yield BMD values. KK was responsible for measurements of bone turnover markers. AY, EK and JSM were responsible for quality control of interview surveys. NO works as a secretary-general of the Administrative Center for the Fujiwara-kyo study. NK, the Principal Investigator of the Fujiwara-kyo study and the Chairperson of the Core Member Committee, conceived of and designed the Fujiwara-kyo study, and participated in designing the FORMEN study. All authors critically revised the manuscript and approved the final version of the manuscript for publication.

## Pre-publication history

The pre-publication history for this paper can be accessed here:

http://www.biomedcentral.com/1471-2474/10/165/prepub
